# The Effect of Exercise on Visceral Adipose Tissue in Overweight Adults: A Systematic Review and Meta-Analysis

**DOI:** 10.1371/journal.pone.0056415

**Published:** 2013-02-08

**Authors:** Dirk Vissers, Wendy Hens, Jan Taeymans, Jean-Pierre Baeyens, Jacques Poortmans, Luc Van Gaal

**Affiliations:** 1 Faculty of Medicine and Health Sciences, University of Antwerp, Antwerp, Belgium; 2 Health Department, Bern University of Applied Sciences, Berne, Switzerland; 3 Faculty of Physical Education and Physiotherapy, Vrije Universiteit Brussel, Brussels, Belgium; 4 Higher Institute of Physical Education and Physical Therapy, Université libre de Bruxelles, Brussels, Belgium; 5 Department of Endocrinology, Diabetes and Metabolism, Antwerp University Hospital, Edegem, Belgium; NIDDK/NIH, United States of America

## Abstract

Excessive visceral adipose tissue appears to trigger a cascade of metabolic disturbances that seem to coexist with ectopic fat storage in muscle, liver, heart and the ß-cell. Therefore, the reduction of visceral adipose tissue potentially plays a pivotal role in the treatment of the metabolic syndrome. The purpose of this systematic review and meta-analysis is to describe the overall effect of exercise on visceral adipose tissue and to provide an overview of the effect of different exercise regimes, without caloric restriction, on visceral adipose tissue in obese persons. A systematic literature search was performed according to the PRISMA statement for reporting systematic reviews and meta-analyses. The initial search resulted in 87 articles after removing duplicates. After screening on title, abstract and full-text 15 articles (totalling 852 subjects) fulfilled the a priori inclusion criteria. The quality of each eligible study was assessed in duplicate with “The Critical Review Form for Quantitative Studies”. Using random-effects weights, the standardized mean difference (Hedge's g) of the change in visceral adipose tissue was −0.497 with a 95% confidence interval of −0.655 to −0.340. The Z-value was −6.183 and the p-value (two tailed) was <0.001. A subgroup analysis was performed based on gender, type of training and intensity. Aerobic training of moderate or high intensity has the highest potential to reduce visceral adipose tissue in overweight males and females. These results suggest that an aerobic exercise program, without hypocaloric diet, can show beneficial effects to reduce visceral adipose tissue with more than 30 cm^2^ (on CT analysis) in women and more than 40 cm^2^ in men, even after 12 weeks.

## Introduction

Obesity (BMI ≥30.0 kg.m^−^^2^) affects more than one third of Americans and a fifth of Europe's population. The prevalence of obesity has reached epidemic proportions with rates of severe childhood obesity that have tripled in the last 25 years.[1], [2] Obesity is associated with an increased risk of comorbidities and increased risk of premature death.[3], [4] Over the last several years, increasing attention has been paid to abdominal adiposity and its association with increased mortality.[5], [6] Visceral adipose tissue (VAT) seems to be the most pathogenic fat depot and is considered to play a central role in the metabolic syndrome. Adipose tissue is not only considered an energy storage organ but is now recognized as an endocrine and paracrine organ that plays an active role in energy homeostasis through the release of a large number of cytokines and bioactive mediators.[7] These novel risk factors and markers can not only influence body weight homeostasis but also insulin resistance, diabetes, lipid metabolism, inflammation, explaining premature atherosclerosis in obesity.[8] This has caused the hypothesis on the underlying mechanisms of the metabolic syndrome to shift towards an adipose tissue disease (adiposopathy) or lipotoxicity.[9] Exercise plays a key role in the prevention and treatment of overweight[10] and the non-pharmacological treatment of dyslipidemia.[11] It is also well established that participation in regular physical activity improves blood glucose control and can prevent or delay type 2 diabetes [12] and has the potential to reduce blood pressure.[13] Evidence for the positive effect of exercise on novel risk factors of the metabolic syndrome such as disturbances in adipokine secretion and low-grade inflammation confirms the importance of exercise in the treatment of the ‘new concept’ metabolic syndrome.[14] Reduction of body weight by lifestyle intervention is often modest, thus therapies which give the best result in decreasing VAT should be favoured over, or combined with, others. In keeping with earlier findings that reported the need for a higher volume on RCT's on the influence of physical activity in abdominal fat[15], in this review literature was screened to determine the effect of different exercise regimes (without hypocaloric diet) on visceral adipose tissue in overweight and obese adults.

## Methods

The systematic literature search was performed according to the Preferred Reporting Items for Systematic Reviews and Meta-Analyses (PRISMA) statement [16].

### Study selection

Any randomized (RCT) and non-randomized controlled trials (non-RCT) or clinical trials (CT) meeting the subsequent specifications were included. Trials were included if the mean age of participants (males and females) was older than 18 years (adults, no upper age limit) and if the mean BMI at baseline was above 25.0 kg.m^−2^. Studies with one or more cohorts participating in aerobic or resistance exercise (physical activity) were eligible for inclusion in the meta-analysis. Physical activity was defined as a program that included voluntary aerobic or resistance exercise at “low to moderate” or “vigorous” intensity for at least two sessions per week. Study inclusion was limited to four discrete measurements of visceral adipose tissue: 1) Computerized Tomography (CT scan), 2) Magnetic Resonance Imaging (MRI), 3) dual energy x-ray absorptiometry (DXA), and 4) Ultrasound (US). These tests were selected because of their documented validity and reliability for assessments as well as reported prevalence in the literature.[17], [18] The studies were expected to conduct a (supervised) physical activity intervention without dietary intervention and to include all information needed for further meta-analysis in order to be considered for inclusion. Diet-only and supplementation-only studies or studies not meeting the inclusion criteria (e.g. physical activity intervention less than 8 weeks) were excluded. If a study examined the effects on visceral adipose tissue in diet and exercise groups, only the data of the exercise intervention arm was included in the final analysis.

### Data sources and search strategies

Databases that were systematically searched were Pubmed, SPORTDiscus, Pedro and Cochrane. The following search strategy was conducted (adapted for each database): (Overweight OR Obesity) AND (Exercise OR “Physical activity” OR “Exercise therapy” OR “Resistance training” OR “Aerobic training”) AND (“Visceral adipose tissue” OR “Intra-abdominal fat”).

Studies published in English, German, French and Dutch were included. The date range was from 1990 to August 2012.

Reference lists were checked for any topic-related relevant studies.

Hand searching and screening for abstracts and citations from annual scientific conferences relating to exercise science were not performed.

The corresponding author of a study was contacted if needed to obtain any missing information or data. If authors could not be reached or if the data were no longer available, the trial was not included in the meta-analysis.

### Screening and data-extraction form

All citations identified by electronic databases were organized and the duplicates were deleted. Initially, two investigators independently screened the results from the electronic searches in order to select potentially relevant citations based on titles and abstracts. The kappa statistic was used to evaluate the chance-adjusted inter-reviewer agreement (Kappa = 0.94). Inter-reviewer disagreements about study eligibility were resolved through consensus. For articles with relevant citations or with titles/abstracts that were not sufficient for deciding on inclusion/exclusion, the full-text articles were retrieved and evaluated. All studies selected at the first screening step were read and abstracted independently by three reviewers. Differences between the reviewers were resolved by consensus or referred to the third reviewer if necessary.

The following study characteristics were extracted from the articles: publication year, journal, study design, BMI, gender, type of intervention, study size, study duration, volume of physical activity, intensity of physical activity and change in VAT. Missing information was requested from authors by email.

### Quality assessment

The quality of each eligible study was assessed in duplicate. Disagreements were resolved by mediation, if necessary with input from a third investigator.

The Critical Review Form for Quantitative Studies (Mc Master University 1998)[16] was used for quality assessment, resulting in a maximum score of 15. Only studies with a score of 8 or higher were included.

### Statistical analysis

A meta-analysis with a random-effects model (specified a priori), accounting for possible heterogeneity between the studies, was used to examine the overall effect size of physical activity on visceral adipose tissue.

Effect sizes (change in VAT) of the uncontrolled and controlled studies were calculated as standardized mean differences and expressed as Hedge's *g* to correct for overestimating the true effect. The 95% confidence intervals [95%CI] were calculated for the individual studies and the overall estimate.

Subgroup analyses were conducted to assess the influence of different co-variates, such as the intensity of physical activity, on the overall estimate of VAT change. Meta regression was used to assess the possible influence of the duration (expressed as weeks) of intervention on the effect sizes of the 15 studies under investigation.

The Cochran's *Q* statistic and *I^2^* were calculated to assess the degree of heterogeneity across studies. Publication bias was assessed using visual analysis of the funnel plot and by formal testing for funnel plot asymmetry using the ‘trim and fill’ and the ‘fail ’n safe' algorithms. For all analyses, *P* values less than 0.05 were considered significant. All calculations and plots were conducted using the CMA-2 software (Comprehensive Meta-Analysis 2^nd^ version, Biostat, Englewood, NJ, USA).

For purpose of clinical interpretation the overall estimate of a meta-analysis on a subgroup of five controlled studies which used the same measurement scale (cm^2^) was re-expressed in the original units following the guidelines as described in the Cochrane handbook for systematic reviews of interventions[19]. Baseline data of the McTiernan study[20] was used to calculate a pooled standard deviation for the female and male experimental and control groups as well as for the combined gender groups.

## Results

### Overview of included studies for the meta-analysis

**Figure 1:**
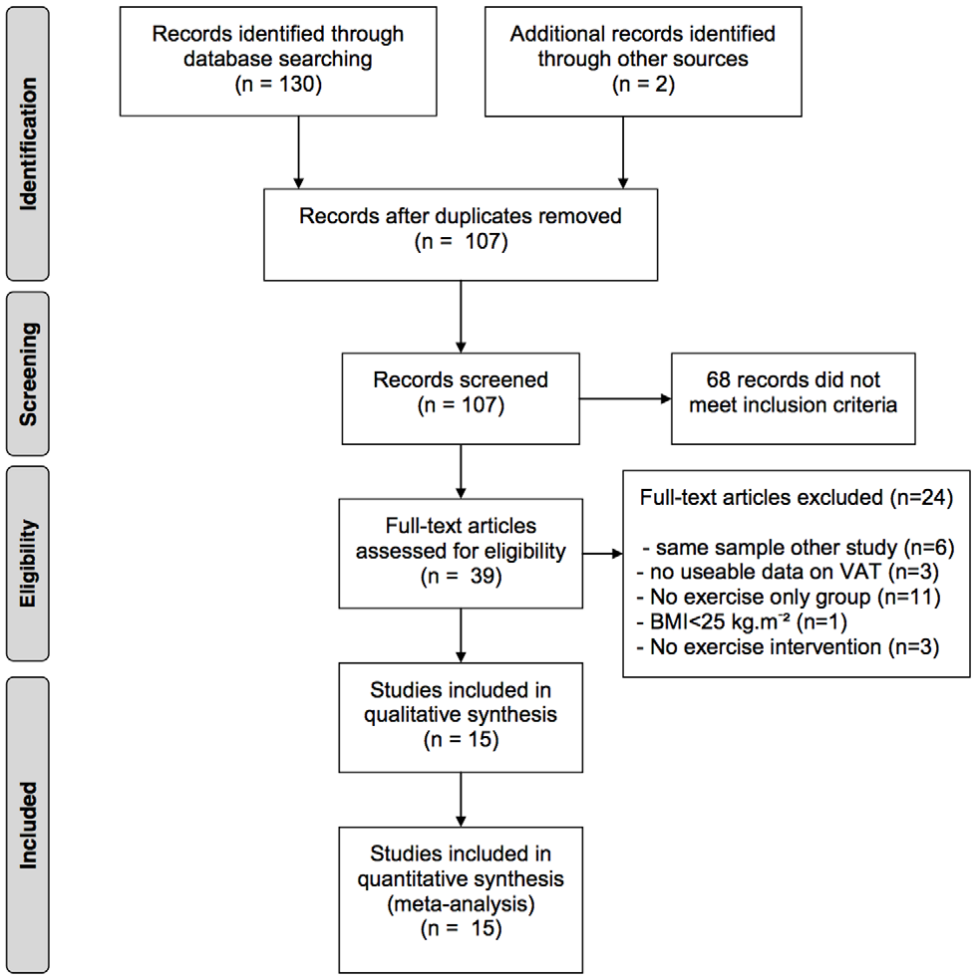
Four-phase flow diagram of the systematic reviewing process.

The initial search resulted in 107 articles after removing duplicates. After screening on title, abstract and full-text 15 articles (totalling 852 subjects in the exercise-only groups) fulfilled the a priori set inclusion criteria (Fig. 1). Because all studies that used dual energy x-ray absorptiometry or ultrasound did not fulfill one or more inclusion criteria, all of the 15 included studies used CT scan or MRI to quantify VAT.

### Study quality

All of the 15 studies included in the qualitative analysis (fig 1) scored more than the a priori defined limit of 8/15 (range = 15 to 10), assessed with the “Critical Review Form–Quantitative studies” assessment instrument. In five of the fifteen studies, the reviewers independently agreed on all the items. For the other studies the degree of disagreement ranged between 1 and 5 items (mostly content validity related items). Disagreement was solved by consensus in all cases.

### Meta-analysis

**Figure 2:**
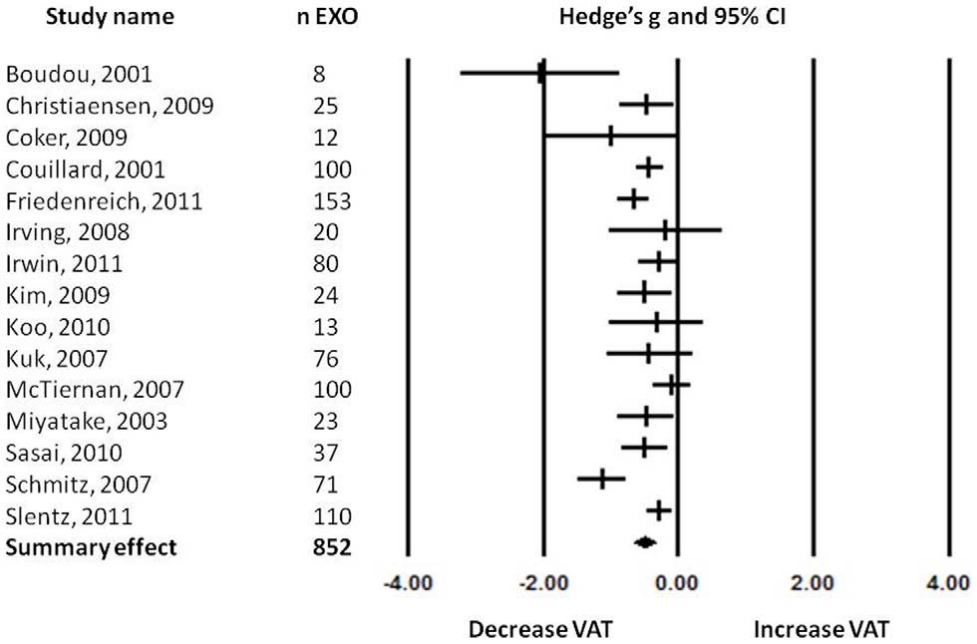
Forest plot of the effects found in the individual studies and the overall effect.

Data were extracted by a reviewer and presented in a spreadsheet to two other reviewers. The data were discussed until consensus was reached and analyzed in work meetings. All fifteen studies analyzed the effect of exercise on visceral adipose tissue (Table 1). The results of these studies were analyzed using the random effects model because of the high heterogeneity between studies (Fig 2). The standardized mean difference (Hedge's *g*) of the change in visceral adipose tissue after a physical activity intervention was −0.497 with a 95% confidence interval (95%CI) of −0.655 to −0.340. The *Z*-value was −6.183 and the *p*-value (two tailed) was <0.001. Heterogeneity analysis showed low to moderate, but significant, heterogeneity between studies (Cochran's *Q* = 37.317; degrees of freedom of *Q* (df(Q)) = 14; *p* = 0.001; *I^2^* = 62.484).

**Table 1:** Overview of the studies included in the meta-analysis.

Studies (Year)	N EXO (M/F)	Age (y)	BMI (kg/m^2^)	Training	Intensity	Frequency	Duration/session	Duration intervention	Assessment VAT	Results VAT
Boudou et al.[21] (2001)	8 (8/0)	45.4±7.2	29.6±4.6	2x/w aerobic1x/w interval	aerobic: 75% VO_2peak_ interval: −5×2 min 85% VO_2peak_−3 min 50% VO_2peak_	3x/week	aerobic: 45 min interval: 25 min	10 weeks	MRI L4–L5	from 153.25 cm^2^ ±38.55 to 84.20 cm^2^ ±21.30
Couillard et al.[51] (2001)	100 (100/0)	High TG/low HDL: 42±14 Isolated high TG: 30±14	HIGH TG/LOW HDL: 27.4±4.4 Isolated high TG: 28.8±4.2	aerobic	55–75% VO2_max_	3/week	50 min	5 months	CT L4–L5	High TG/low HDL: −10.8 cm^2^±21.1 Isolated high TG: −5.0 cm^2^±17.2
Irwin ML et al.[36] (2003)	80 (0/80)	61.0	30.5	combination	60–75% HR_max_	5/week	45 min	12 months	CT L4–L5	−8.5 g/cm^2^
Miyatake et al.[52] (2003)	23 (23/0)	45.2±7.5	29.0±2.3	aerobic	50%–65% HR_max_	1/week and increasing steps with 1000/day	N.A.	5 months	CT at level umbiculus	from 108.7 cm^2^±49.1 to 85.9 cm^2^±40.9
Kuk et al. [53] (2007)	76 (0/76)	On average 58y	On average 31	aerobic	50%VO2_max_	3–4/week	4–8–12 kcal/kg/w	6 months	CT L4–L5	−0.02 kg
McTiernan et al. [20] (2007)	100 (51/49)	F: 54.4±7.1 M: 56.2±6.7	F: 28.9±5.5 M: 29.7±3.7	aerobic	60–85% HR_max_	6/week	60 min	12 months	CT L4–L5	−12.2 cm^2^ (−7.5%)
Schmitz et al. [54] (2007)	71 (0/71)	36±5	29.4±0.4	strength	3×8–10 reps/exercise	2/week	60 min	12 months	CT L2–L3	−2.99%
Irving BA et al.[24] (2008)	20 (0/20)	51±9	34±6	aerobic	low: rpe 10–12 high: rpe 15–17	5/week	300–400 kcal/session	16 weeks	CT L4–L5	High Intensity: from 173 cm^2^±73 to 148 cm^2^±59 Moderate Intensity: no significant changes
Christiansen et al.[55] (2009)	25 (0/25)	37.2±7	34.3	aerobic	70% HR_res_	3/week	60–75 min	12 weeks	Multislice MRI, femur to T8–T9	−18%
Coker et al. [22] (2009)	12 (6/6)	HI: 73±2 MI: 70±1	HI: 30±1 MI: 28±1	aerobic	HI: 75% VO_2peak_MI: 50% VO_2peak_	4–5/week	1000 kcal/week	12 weeks	CT L4–L5	High Intensity: −39 cm^2^±11Moderate Intesity: no change
Kim et al. [37] (2009)	24 (24/0)	49.4±9.6	30.7±3.3	aerobic	60–70% Hrmax	3/week	60 min	12 weeks	CT at level umbiculus	from 197.1 cm^2^ to 165.7 cm^2^ (±16%)
Koo et al. [56] (2010)	13 (0/13)	59±4	28.0±2.7	aerobic	brisk walking±500 kcal/d	7/week	120 min	12 weeks	CT L4–L5	−29,7%±23.3%
Sasai et al.[57] (2010)	37 (37/0)	47.6±8.6	Low Volume: 31.0±4.1High Volume: 29.3±2.0	aerobic	65–80% HR_max_	3/week	60–90 min	12 weeks	CT at level umbilicus	High Volume: −30.0 cm^2^ ±23.4 Low Volume: −17,8 cm^2^±37.5
Friedenreich et al.[23] (2011)	153 (0/153)	61.2±5.4	29.1±4.5	aerobic	62% HR_res_	3.6/week	45 min	12 months	CT at level umbilicus	−16.5 cm^2^
Slentz et al. [45] (2011)	110	ST: 49.7 ±11.4 AT: 49.5 ±9.8 COMB: 46.9 ±10.0	ST: 30.5 ±3.4AT: 30.4 ±3.2COMB: 30.7 ±3.4	strengthaerobiccombination	75% VO_2peak_ (14 kcal/kg/w)	2–3/week	ST: 3 sets/day, 8–12 reps/set, 8 exercisesAT: eq. To 19.2 km/week	8 months	CT at level L4 pedicle	ST: + 0.8 cm^2^ ±19AT: −15.9 cm^2^ ±34COMB: −10.9 cm^2^ ±9

* Sample sizes represent exercise-only groups with results on VAT. ** Statistical significant results (p<0.05) are marked in bold.

**Figure 3:**
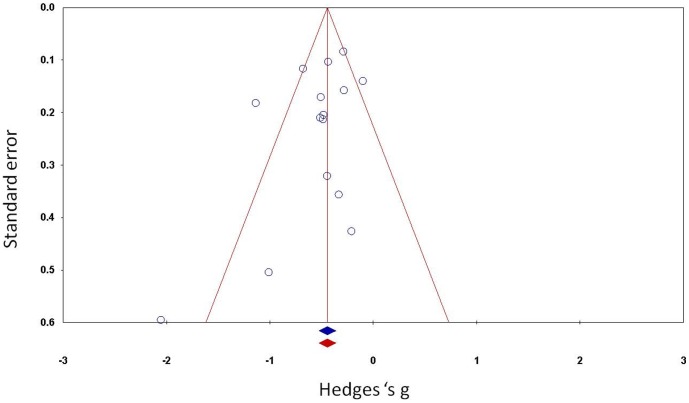
Funnel plot of the standard error by Hedge's g.

Through a funnel plot of standard error by Hedge's *g*, a graphic representation of heterogeneity was obtained (Fig 3). Using the ‘fail’n safe' algorithm, it becomes clear that there was no publication bias in the analysis because 405 extra studies would be needed to get the *p*-value to the alpha level.

Three of the analyzed studies (Boudou 2001, Cooker 2009, Koo 2010) had small sample sizes (*n*<14) and could thereby overestimate the general effect of the therapy. Conducting a sensitivity analysis by removing these studies, still revealed a decrease in visceral adipose tissue of −0.464 Hedge's *g* after a physical activity intervention program (95%CI: −0.313 to −0.616, *p*<0.01). By making this advanced analysis, heterogeneity decreased, however only slightly, to Q  =  28.741 (with *p* = 0.002, *I^2^* = 61.727).

### Subgroup analysis and Meta-Regression

#### Controlled versus uncontrolled

**Figure 4:**
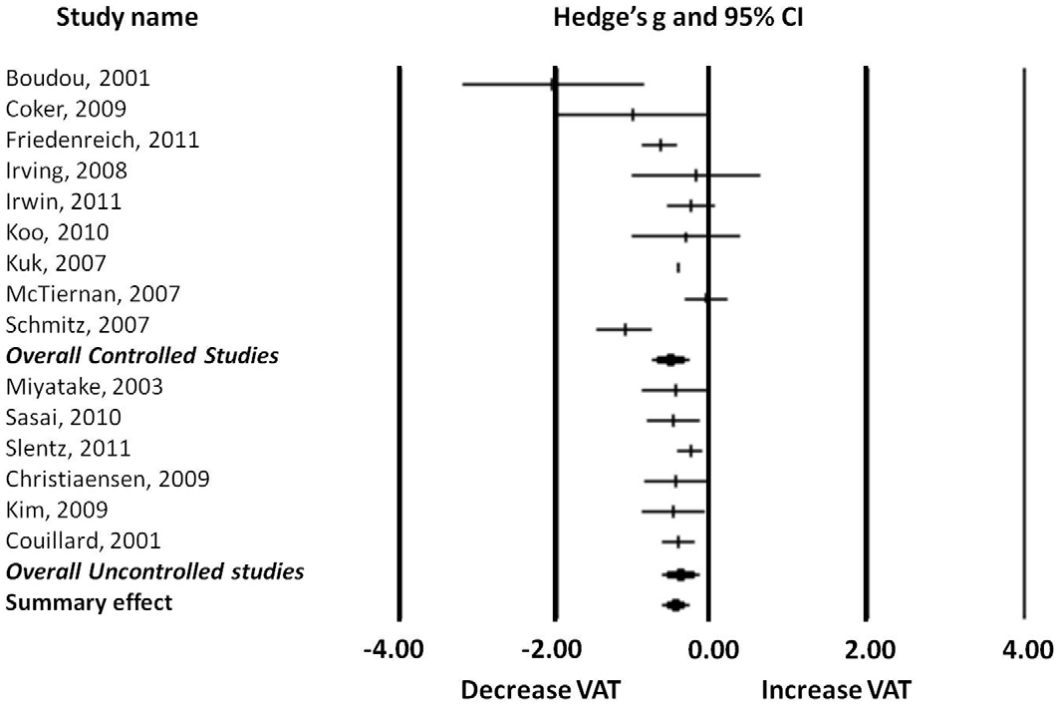
Forest plot of the subgroup analysis: controlled and uncontrolled studies.

For the first subgroup analysis, the studies were divided in nine controlled and six uncontrolled trials (Fig 4). The analysis of these subgroups revealed that the controlled studies found a greater effect when comparing the results of VAT of the control group with those of the intervention group (Hedge's *g* = −0.561, 95%CI: −0.332 to −0.791, *p*<0.01) than uncontrolled studies in which the effect was compared between pre and post exercise intervention VAT values (Hedge's *g* = −0.437, 95%CI: −0.207 to −0.667, *p*<0.01).

Heterogeneity analysis showed significant moderate to high heterogeneity between controlled studies (Cochran's *Q* = 32.888; df(*Q*) = 8; *p*<0.001; *I^2^* = 75.675) but homogeneity between uncontrolled studies (Cochran's Q  =  2.469; df(Q)  =  5; *p* = 0.781; *I^2^* = 0.000).

#### Gender

In female studies the decrease in VAT was −0.550 Hedge's *g* (95%CI: −0.269 to −0.831, *p*<0.01) while in male studies the decrease in VAT was −0.589 Hedge's *g* (95%CI: −0.276 to −0.896, *p*<0.001). In two studies where there were mixed samples of males and females, a lower overall decrease of VAT was observed. (Hedge's *g* = −0.330, 95%CI: −0.045 to −0.706, *p* = 0.085). Heterogeneity analysis showed significant low to moderate heterogeneity in mixed (Cochran's *Q* = 10.021; df(*Q*)  =  4; *p* = 0.040; *I^2^* = 60.085), male (Cochran's Q = 22.007; df(*Q*) = 6; *p* = 0.001; *I^2^* = 72.736) and female studies (Cochran’s Q = 25.878; df(*Q*) = 8; *p* = 0.001; *I^2^* = 69.086).

By removing the data of the subjects who received strength training or mixed (aerobic and strength) training, heterogeneity between studies decreased, especially in female and mixed gender study populations (Cochran’s Q = 13.279; df(*Q*) = 6; *p* = 0.039; *I^2^* = 54.815 vs. Cochran’s Q = 6.113; df(*Q*) = 2; *p* = 0.047; *I^2^* = 67.282). The effect of physical activity stayed significant in both male (Hedge’s *g* = −0.545, 95%CI: −0.286 to −0.805, *p*<0.001) and female studies (Hedge’s *g* = −0.334, 95%CI: −0.083 to −0.585, *p*<0.001). However, in the mixed study groups, the effect of physical activity did not reach significance (Hedge’s *g* = −0.396, 95%CI: −0.054 to −0.847, *p* = 0.085).

#### Training Modality

All studies were categorized in “aerobic training”-studies, “strength training”-studies or “combined” subgroups. This subgroup analysis showed that the decrease in VAT in the aerobic training subgroup was higher (Hedge’s *g* = −0.550, 95%CI: −0.332 to −0.768, *p*<0.001) compared to the strength training subgroup (Hedge’s *g* = −0.529, 95%CI: −0.003 to −1.054, *p* = 0.049).

However, in the “combined” subgroups, the decrease in VAT was no longer significant (Hedge’s *g* = −0.301, 95%CI: 0.221 to −0.823, *p* = 0.258).

Heterogeneity between aerobic studies was significant low to moderate (Cochran’s *Q* = 39.121; df(*Q*) = 16; *p* = 0.001; *I^2^* = 59.101) while significant low to high heterogeneity was observed between strength studies (Cochran’s *Q* = 23.824; df(Q) = 1; *p*<0.001; *I^2^* = 95.803). Homogeneity was found between combination studies (Cochran's *Q* = 0.034; df(*Q*) = 1; *p* = 0.853; *I^2^* = 0.000).

#### Training Intensity

**Figure 5:**
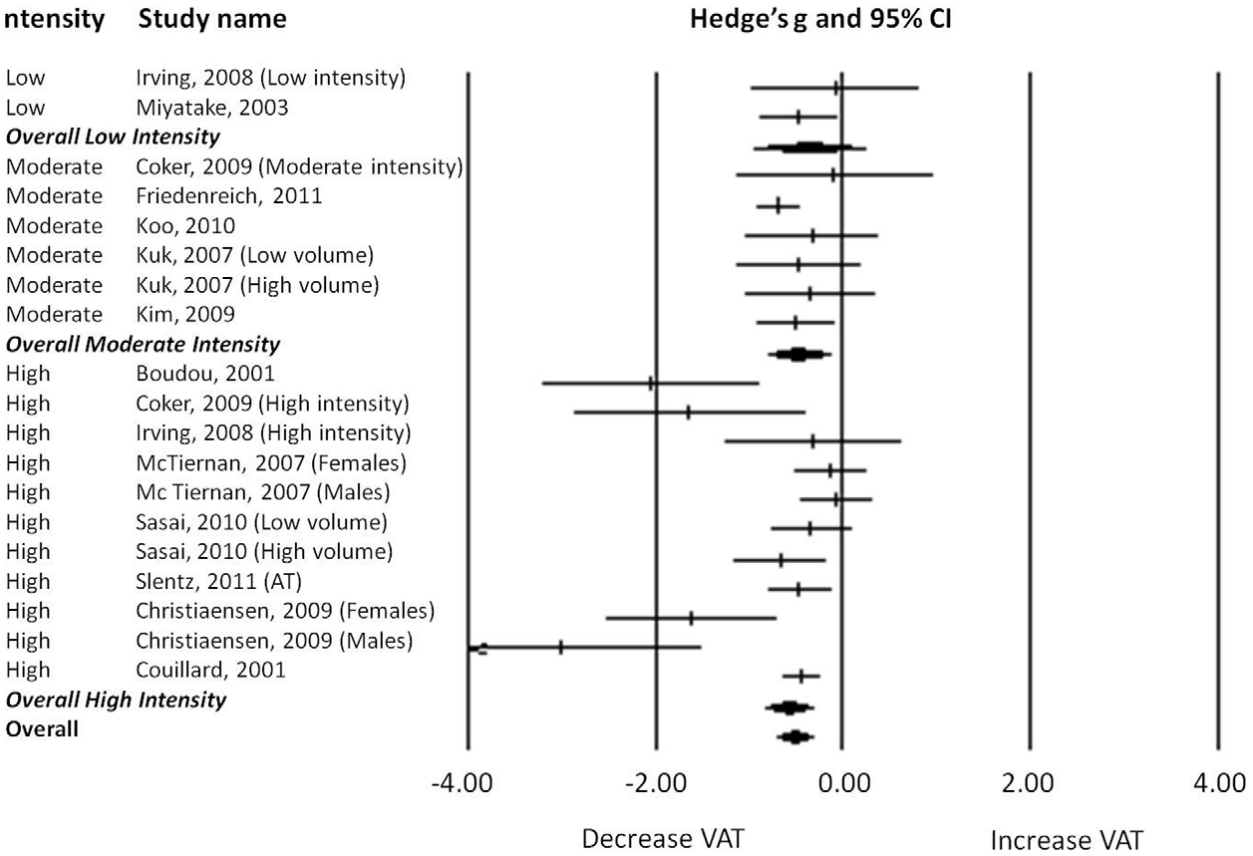
Forest plot of the subgroup analysis: low intensity, moderate intensity and high intensity studies.

A final subgroup analysis was made through the categorization for intensity of all aerobic studies in “high intensity”, “moderate intensity” and “low-intensity” studies (Fig 5). Following cut-off values were used: 1) “high intensity”: when training most of the time at an intensity≥70% of maximal heart rate (HRmax) or more than 55% of maximal oxygen consumption (VO2max) or 60–80% of the heart rate reserve (HRres); 2) “moderate intensity”: when training most of the time at an intensity between 60–70% HRmax or 45−55% VO_2_max and 3)“low intensity”: when training most of the time at an intensity<60% HRmax or <45% VO_2_max.

This analysis showed that a certain threshold of physical activity intensity should be reached to obtain a decrease in VAT. Only in studies in which moderate and high intensity-training was used, the effect on VAT was significant (“moderate intensity”-training: Hedge's *g* = −0.473, 95%CI: −0.140 to −0.806, *p* = 0.005; “high intensity”-training: Hedge's *g* = −0.588, 95%CI: −0.336 to −0.840, *p*<0.001).

Heterogeneity analysis showed significant low to moderate heterogeneity between “high intensity”-studies (Cochran's *Q* = 35.524; df(*Q*) = 10; *p*<0.001; *I^2^* = 0.144) and homogeneity between “low intensity”-studies (Cochran's *Q* = 0.627; df(*Q*) = 1; *p* = 0.428; *I^2^* = <0.001) and “moderate intensity”-studies (Cochran's *Q* = 2.659; df(*Q*) = 5; *p* = 0.751; *I^2^* = <0.001).

The regression model showed no significant effect of duration of the intervention on VAT decrease ( = 0.836)

#### Re-expression of the estimate for clinical purpose

The five controlled studies (totalling 569 participants) using cm^2^ as the measuring scale of VAT[20]–[24] showed an overall estimate of Hedge's *g* = −0.630 representing a pooled effects size of −37.1 cm^2^ (females), −46.5 cm^2^ (males) and −42.1 cm^2^ (combined gender groups) respectively.

## Discussion

### Summary of findings

The present systematic review and meta-analysis is the first to investigate the effect of exercise without diet on visceral adipose tissue specifically in overweight and obese adults, with subgroup analyses for gender and intensity. All studies included in the meta-analysis used CT scan or MRI to assess VAT. There seems to be a strong association between visceral adipose tissue and increased risk for diseases such as cardiovascular disease[25], [26], type 2 diabetes[27] and non-alcoholic fatty liver disease[28]. A Cochrane review[29], states that a strong association exists between physical activity and improved cardiovascular disease risk factors, independent of weight reduction. Reduction of visceral adipose tissue may play a pivotal role in the pathophysiological mechanisms of this association.[30] Therefore, it is of great clinical interest to know if exercise is suited to reduce VAT because this could be an important clinical target, independent of (large) weight loss, which is often difficult to achieve and maintain.

This meta-analysis showed that a decrease of visceral adipose tissue can be obtained by exercise without diet in people with overweight and obesity. Aerobic exercise of moderate to vigorous intensity seems to have a greater effect on VAT than low intensity aerobic exercise or strength training. This is in line with the findings of Ismael et al.[31] who concluded in their meta-analysis that aerobic exercise is central for exercise programs aimed at reducing VAT. The study of Ismael et al. included predominantly studies with overweight and obese participants, but also to some extent studies with normal weight participants. Differences between the present meta-analysis and the meta-analysis of Ismael et al. are that the present meta-analysis 1) focused exclusively on overweight and obese individuals, 2) only included exercise-only intervention groups, 3)required a minimum of 8 weeks of exercise for inclusion, 4) presented results of subgroup analyses for gender and training intensity, 5) included studies published up to August 2012 and 6) re-expressed Hedge's g in cm^2^ for clinical interpretation.

Despite the large heterogeneity between studies, demonstrated by the sensitivity analyses, an overall effect of physical activity was found in this meta-analysis. Heterogeneity could possibly be explained by the diversity in study designs, training protocols and characteristics of subjects (age, BMI, gender, randomization,...). As demonstrated by Okura et al. abdominal fat reduction in response to weight loss can be modified by obesity phenotype (intra-abdominal versus abdominal subcutaneous fat storage).[32] The diversity of obesity phenotypes could also play a role in the heterogeneity that was found in this meta-analysis. Also ethnicity or race can play a role in obesity phenotype and more specifically in visceral adipose tissue storage[33], [34] In this meta-analysis most studies had Caucasian or Asian participants. A subgroup analysis revealed no significant difference in reduction of VAT between these 2 groups (results not shown).

### Training volume

As described in the 2009 position stand of the American College of Sports Medicine (ACSM), moderate-intensity physical activity of >250 min.wk^−1^ has been associated with clinically significant weight loss.[35]

In the studies of McTiernan et al.[20] and Irwin et al.[36], participants trained five to six times per week for 45 to 60 minutes, which was twice as much as in the studies of Friedenreich et al.[23] and Kim et al[37]. Surprisingly, the higher training volume didn′t result in a higher reduction of VAT or total fat percentage. This is in contrast with the findings of Friedenreich et al.[23] who concluded that a minimum dose of physical activity must be achieved to yield a reduction in VAT while high volume training programs resulted in a higher reduction of VAT. The totally or partially self reported adherence to the exercise programs using activity diaries in the McTiernan et al.[20] and Irwin et al.[36] studies may have confounded the results by an adherence overestimation.

### Training intensity

In the subgroup analysis for training intensity, only the studies with an aerobic exercise-only protocol were included. The results showed that there seems to be a threshold for intensity in order to have an effect on the reduction of VAT.

Although both absolute and relative (i.e.% of VO_2_max) exercise intensities play important roles in the regulation of substrate metabolism, the relative exercise intensity plays a major role in determining the proportions of carbohydrate and fat oxidized by the working muscles.[38] However, individual characteristics, such as nutritional status, may explain a large part of the variation in maximal rates of fat oxidation during exercise.[39] Also the fact that increasing exercise intensity can increase postexercise energy expenditure and fat oxidation should be taken into account.[40] The cut-offs to classify intensity in relation to a decrease in adipose tissue, as used in this meta-analysis, remain therefore somewhat arbitrary. An association has been reported between the volume of physical activity and weight loss[41], with indications for a possible ‘dose-response relationship’ between exercise intensity and increase in lean body mass. Also an association between cardiorespiratory fitness and diminished abdominal adiposity has been described[42] and between the amount of physical activity and the risk for metabolic syndrome[43]. However, the direct association between exercise intensity and reduction in visceral fat has not been investigated as a primary goal extensively. Gutin et al.[44] reported no clear effect of the intensity of physical training on the reduction of visceral and total-body adiposity. The study of Irving et al.[24] is one of few studies to report on the effect of exercise intensity in obese adults with abdominal visceral fat as primary outcome parameter.

### Gender

There were an equal number of male and female studies. Only two mixed gender studies (Coker et al.[22]; Slentz et al.[45]) fulfilled the a priori set inclusion criteria, making it difficult to generalize the findings of this subgroup analysis. The analysis results suggested that in mixed gender studies, the effect of physical activity on VAT is smaller. Participants in those two studies showed only a minor loss of body weight while their reduction in relative body fatness was not mentioned.

This meta-analysis confirmed the need for gender-specific approaches and outcomes of obesity treatment in general, as previously stated by Lovejoy et al.[46] and more specific in the treatment of abdominal obesity. Furthermore, the results of this meta-analysis showed that males yield a higher profit of exercise on VAT than women corroborating the findings of Redman et al.[47]. The latter found more effect of caloric restriction and of the combination of caloric restriction and aerobic exercise in men then in women. In this context, one should take into account the facts that obesity phenotype can have an impact on abdominal fat reduction[32] and that men tend to be more likely to have significant amounts of abdominal fat, and to be more susceptible to abdominal adiposity[48].

### Aerobic exercise and strength training

Only two combined aerobic and strength training studies fulfilled the inclusion criteria (Irwin et al.[36]; Slentz et al.[45]). The meta-analysis showed that such a combination yields only a modest reduction in VAT. This is somewhat unexpected because both aerobic training and strength training separately proved to have a significant effect on reduction of VAT. In the study of Slentz et al.[45], the combined training was not found to be superior to the aerobic training but the effects of both training types were similar.

### Study strengths and limitations

A structured study protocol was developed and utilized for the search strategy, study selection, data extraction and statistical analysis. When the description of the methods was vague or insufficient data were given, the corresponding authors were contacted. All 15 included studies received scores of 10 or higher on the “Critical Review Form–Quantitative studies” and could thereby be considered to be of sufficient methodological quality.

Despite of all efforts, there are some limitations of this review that need to be acknowledged. In the assessment of the quality, the items with the highest disagreement between both scorers were “detailed description of the sample”, “justifying of the sample”, “avoiding of contamination” and “avoiding of co-intervention”. Only in a few studies, a power measurement of the sample had been done and often recruitment of the sample was not specified. There were also few studies that objectively assessed adherence to the exercise program and nutritional intake.

The strengths of this meta-analysis are that it focuses on the effects of exercise specifically in overweight and obese adults and provides information based on subgroup analyses for gender and training intensity. In a relentless effort to make standardized mean differences more clinical interpretable, Hedge's g were re-expressed as units of VAT, more specifically cm^2^.

Based on the Hedge's g, it seems that the 5 controlled clinical trials that used cm^2^ as unit for VAT, slightly overestimate the effect of exercise on reduction of VAT compared to the total of 9 controlled clinical trials (−0.630 versus −0.561). Taking that into account, the results of this meta-analysis show that exercise without diet has the potential to reduce VAT with >30 cm^2^ in females and >40 cm^2^ in males. Okauchi et al. demonstrated in a study in 2336 Japanese men that a reduction of VAT within 1 year was associated with a significant decrease in the number of metabolic risk factors, with a decrease of >30 cm^2^ showing the highest correlation coefficient.[49]

The results of the meta-regression of the duration of intervention on the decrease of VAT, seem to suggest that the effect size does not increase by prolonging the intervention period beyond 12 weeks. However, it remains unclear what the effect of the duration of intervention is on the risk of VAT regain. Regain of VAT during the follow-up period after an initial loss of VAT is clearly a pitfall, as demonstrated by Koga et al.[50], which can be predicted by fluctuation (not absolute values) in daily exercise regimens, during the training-education period.

## Conclusion

This systematic review and meta-analysis shows that an exercise program without hypocaloric diet has the potential to reduce visceral adipose tissue.

There seem to be gender differences in decrease of visceral adipose tissue by exercise which could be related to obesity phenotype.

Combining aerobic training with strength training does not result in a higher decrease of visceral adipose tissue. The intensity of a training program should be at least moderate to vigorous. Recommendations for future studies are to take possible confounding factors (such as gender, obesity phenotype, training intensity, type of training) into account and to carefully assess adherence to the training program and nutritional protocol.

## Supporting Information

Table S1 Extended data table including ethnicit and co-morbidities.
(XLSX)

Table S2 Prisma checklist.
(DOCX)
